# Cumulative live birth rates for low-prognosis women over 5 years or 9 frozen-thawed embryo transfer cycles

**DOI:** 10.1186/s12884-022-04511-7

**Published:** 2022-03-22

**Authors:** Di Chen, Xi Shen, Li Wang, Yanping Kuang

**Affiliations:** Department of Assisted Reproduction, Shanghai Ninth People’s Hospital Affiliated to Shanghai Jiao Tong University School of Medicine, 639 Zhizaoju Rd, Shanghai, 200011 China

**Keywords:** Cumulative live birth rate, Low prognosis, Poor ovarian response, POSEIDON criteria, Freeze-all strategy

## Abstract

**Background:**

For heterogeneous populations of low-prognosis women, it remains unclear as to how long individuals should continue undergoing ART when attempting to have a baby, as there have been insufficient studies to date tracking the cumulative live birth rates (CLBRs) for these women over the entire course of their ART treatment, particularly over extended time periods.

**Methods:**

This was a retrospective analysis of 17,698 women at a tertiary care academic medical center who had begun undergoing IVI/ICSI cycles using a progestin-primed ovarian stimulation (PPOS) approach between January 2013 and January 2019. Low-prognosis patients were stratified into four groups based upon POSEIDON criteria, with patients exhibiting normal or high ovarian reserves and response to stimulation (defined as AFC ≥5, > 9 oocytes retrieved) being included as controls (group 5). The CLBR within 5 years or 9 FET cycles from the ovum pick-up (OPU) day of the first cycle was the primary endpoint for this study, including all repetitive oocyte retrieval cycles and subsequent FET cycles. Optimistic and conservative approaches were used for the analysis of CLBRs and the depiction of cumulative incidence curves.

**Results:**

Under both optimistic and conservative model analyses, normal and good responders exhibited the highest CLBR within 5 years or 9 FET cycles, followed by younger unexpected poor responders, younger expected poor responders, older unexpected poor responders, and older expected poor responders. Upward trends in CLBRs were evident across the five groups with the prolongation of time or an increase in FET cycle counts. Within the first 2 years or 3 FET cycles, the CLBRs rose rapidly, followed by more moderate increases over the following 2–3.5 years or 4–6 cycles, with expected poor responders exhibiting the most obvious improvements. All Patients reached a CLBR plateau after 3.5 years or 6 FET cycles.

**Conclusions:**

All low-prognosis women should undergo ART treatment for a minimum of 2 years or 3 FET cycles, and exhibit better outcomes when extending ART treatment to 3.5 years or 6 FET cycles (particularly for POSEIDON groups 3 and 4), but should consider ceasing further treatment thereafter due to a lack of apparent benefit.

## Background

Low prognosis is a concept derived from the poor ovarian response (POR) classification that seeks to better differentiate between POR patients and those with a diminished ovarian reserve (DOR). POR patients undergoing assisted reproductive technology (ART)-based interventions typically exhibit low ovarian reserves and poor ovarian responses to exogenous gonadotropin stimulation, together with high cancellation rates and low rates of resultant pregnancy [1, 2]. In most cases, researchers focus on assessing ART clinical outcomes including odds of pregnancy and delivery on a per-cycle or per-transfer basis [3], and these approaches are not well-suited to comprehensively evaluating outcomes among POR patients that have undergone repeated oocyte retrieval cycles in order to store sufficient embryos for subsequent transfers [4]. The most important outcome for POR patients is the overall chance of a live birth after multiple attempts. As such, the cumulative live birth rate (CLBR) [3, 5], which can track long-term clinical outcomes, has been proposed to be the optimal metric for use when evaluating clinical results associated with the entirety of an ART treatment course.

Previous studies reporting CLBRs have been subject to several limitations. Some have failed to include in vitro fertilization (IVF) cycles involving frozen embryo transfer [6–8], while others have failed to report live-birth rates [7, 9], including multiple deliveries [10–12], as their primary outcomes. Moreover, other studies have calculated cumulative success rates over multiple IVF cycles by adding together rates from different cycles [13, 14]. Currently, CLBR values for a given patient are typically calculated from their first ovarian stimulation, and these rates include the subsequent fresh embryo transfer (ET) and/or frozen embryo transfer (FET) necessary to achieve a minimum of one live fetus as an outcome [3, 15–23]. These criteria are increasingly relevant given the growing frequency of freeze-thaw transfer approaches [3]. To date, however, there have been few studies exploring CLBRs among low-prognosis women undergoing a ‘freeze-all’ transfer strategy [16], particularly over extended periods of time. The present study was thus conducted to offer a more accurate, evidence-based approach to estimating the odds that POR women will experience a live birth when undergoing ART.

The heterogeneity of POR patient populations poses a challenge to diagnosing and treating these individuals [24, 25]. While POR patients were classified according to the first consensus diagnostic criteria - the “Bologna criteria” - by the European Society of Human Reproduction and Embryology (ESHRE) in 2011 [26], many patient categories with potentially different prognostic outcomes may arise within these minimum criteria [27–30]. As such, in 2015, the POSEIDON (Patient-Oriented Strategies Encompassing Individualized Oocyte Number) group introduced a new system for the stratification of infertility patients exhibiting expected or unexpected responses to exogenous gonadotropins [31, 32]. These POSEIDON criteria stratify POR patients according to heterogeneous characteristics, spurring a shift away from POR terminology and towards the concept of low-prognosis patients [31], allowing for the more precise classification of patients according to different diagnostic criteria and the mapping of optimal treatments for different patient subpopulations [33].

In the present retrospective study, low-prognosis patients undergoing a PPOS protocol combined with a freeze-all strategy were grouped according to POSEIDON criteria, and CLBRs within 5 years or 9 FET cycles were compared among patients in different POSEIDON groups. The goal of this study was to determine the odds that a low-prognosis woman will achieve a live birth when undergoing ART and at what point those women should cease undergoing ART attempts. Optimistic and conservative analyses were employed to compare CLBRs among these four POSEIDON groups for up to 5 years or 9 FET cycles, with the goal of formulating individualized treatment recommendations based on analyses of a large population of low-prognosis women undergoing ART treatment.

## Methods

### Study setting and patients

This retrospective study was conducted at the Department of Assisted Reproduction of the Ninth People’s Hospital of Shanghai Jiao Tong University School of Medicine, and included 17,698 women. The study protocol was approved by the hospital’s Ethics Committee (Institutional Review Board). Included patients underwent an initial IVF / intracytoplasmatic sperm injection (ICSI) cycle using a PPOS strategy between January 2013 and January 2019. All patient cycles using a PPOS cycle were followed through August 2020 or until a live birth had been achieved. Low-prognosis patients were categorized into 4 groups according to their age, antral follicle count (AFC), and oocytes retrieved in the first cycle as per the POSEIDON criteria as follows: Group 1 (younger unexpected poor responders): age < 35 years, normal ovarian reserve (AFC ≥ 5), ≤ 9 oocytes retrieved after standard ovarian stimulation in the first cycle; Group 2 (older unexpected poor responders): age ≥ 35 years, normal ovarian reserve (AFC ≥ 5), ≤ 9 oocytes retrieved after standard ovarian stimulation in the first cycle; Group 3 (younger expected poor responders): age < 35 years, poor ovarian reserve (AFC < 5); and Group 4 (older expected poor responders): age ≥ 35 years, poor ovarian reserve (AFC < 5) [31]. Patients with normal or high ovarian reserves and responses to stimulation (AFC ≥ 5, retrieved oocytes > 9) [34] were enrolled as a control group (group 5). Women that underwent treatment with donor semen or that did not undergo subsequent frozen embryo transfers were excluded from this study.

### PPOS and frozen-embryo transfer protocols

Patients were administered hMG (150-225 IU; Anhui Fengyuan Pharmaceutical Co, China) and oral *P* from MC3 onward. Oral *P* included medroxyprogesterone acetate (MPA, 4-10 mg/d, Beijing Zhong Xin Pharmaceutical, China), Utrogestan (200 mg/d, Laboratories Besins International, Paris, France), and oral Duphaston (DYG, 20 mg/d, Abbott Biologicals B.V., Netherlands) [35–38]. The dosage of P was adjusted based upon ultrasound examinations and analyses of serum hormone levels on the same day as follicular monitoring. When more than three dominant follicles (≥ 18 mm in diameter) were evident, the final stage of oocyte maturation was triggered by a single use of hCG (5000–10,000 IU, Lizhu Pharmaceutical Trading Co.) or a dual trigger consisting of a low dose of hCG (1000 IU) and Decapeptyl (0.1 mg, Ferring International Center SA, Germany) [37–39].

Transvaginal ultrasound-guided oocyte retrieval was conducted 35–39 h after trigger [40]. All follicles > 10 mm in diameter were aspirated, with fertilization being achieved via conventional IVF or ICSI, depending on semen quality [41]. As per Cummins’ standard [42], good quality embryos were defined as grade I and II embryos with at least 8 cells on day 3 after oocyte retrieval. Other embryos (non-top-quality embryos) were cultured to the blastocyst stage, with good-quality embryos and the good morphology blastocysts being selected for vitrification [39].

Endometrial preparation was conducted as in prior reports [43]. Briefly, natural cycles were employed for patients with a regular menstrual cycle, while hormone therapy or stimulation cycles were employed for patients with irregular menstrual cycles [44]. One or two embryos were transferred per cycle, with progesterone supplementation being maintained through 8 weeks if pregnancy was confirmed.

### Statistical analysis

The CLBR of patients within 5 years from their OPU day in the first cycle was the primary outcome for the present study, including all repetitive oocyte retrieval cycles and subsequent FET cycles [16]. Baseline characteristics were analyzed based upon data during the first oocyte retrieval cycle for each patient conducted using a progestin-primed ovarian stimulation (PPOS) protocol in our center. Ongoing pregnancies were assessed at 4 and 6 weeks after FET by ultrasound-mediated gestational sac and fetal heartbeat detection. Live birth was defined as any birth event in which at least one baby was born alive [18]. When patients achieved multiple live births, only the first conception event was considered for the present study. Cumulative incidence curves were utilized to assess CLBR by time and FET cycles [45].

To account for ART treatment discontinuation, we employed two evaluation approaches [17, 45]. The optimistic analysis approach assumed that patients who stop treatment would have the same chances of pregnancy as those who continue treatment. This approach, however, has the potential to overestimate the true odds of conception following multiple successive IVF treatments. The conservative competing risk approach, in contrast, assumed that patients who discontinue ART treatment would have a live-birth rate of zero. The actual values will inevitably fall between these two extremes [16, 46]. Differences between these groups were compared with an adjusted pairwise log-rank test.

SPSS v23.0 (SPSS Inc., IL, USA) was used to conduct all statistical analyses. Chi-squared tests and one-way ANOVAs were used to compare categorical and continuous variables, respectively. Patient characteristics are given as percentages or means with standard deviations (SD). *P* < 0.05 was the threshold of significance. CLBR curves with 95% confidence intervals (95% CIs) were calculated using R (v 1.4.1106; R Foundation for Statistical Computing, Vienna, Austria).

## Results

### Patient demographics and baseline characteristics

**Fig. 1:**
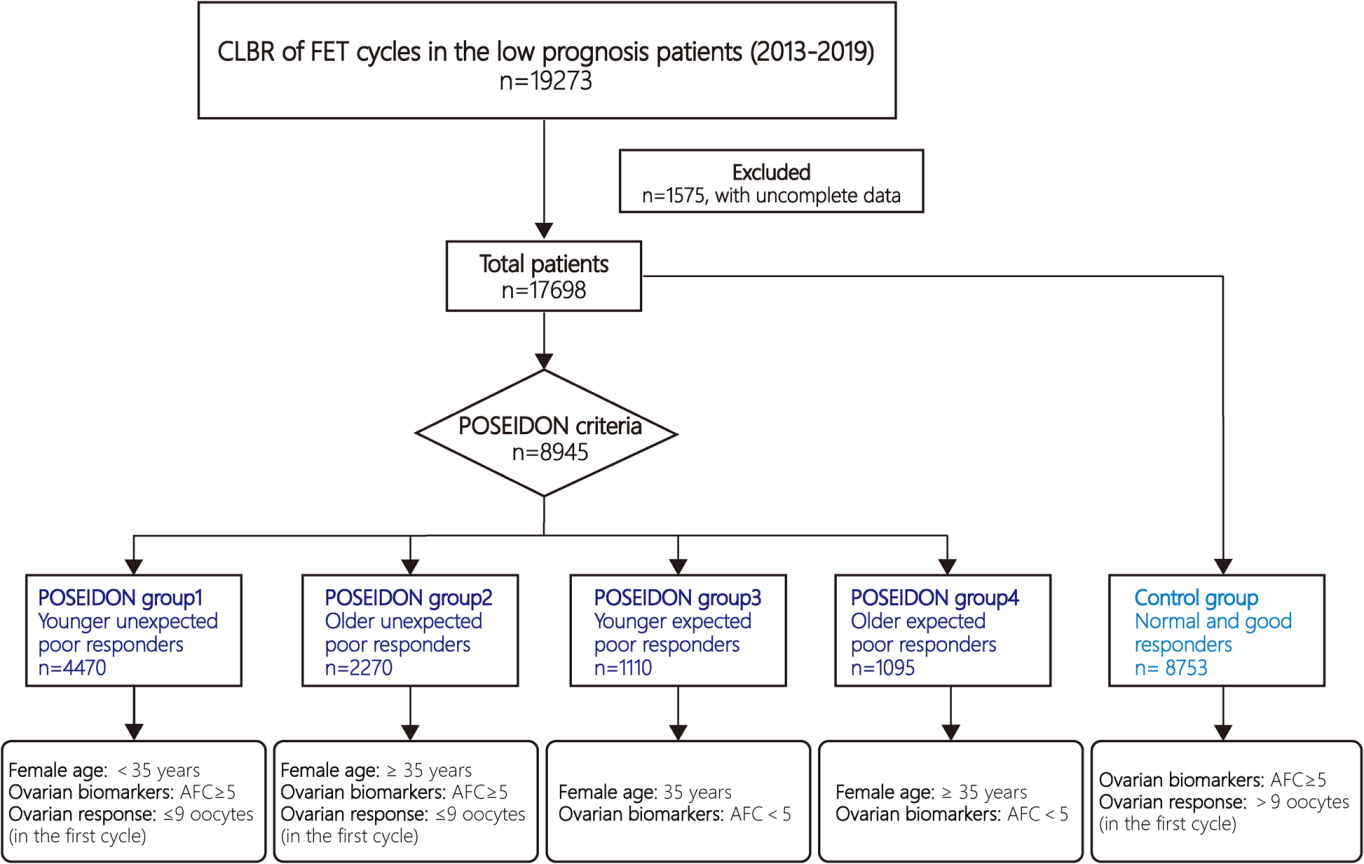
Study population flow chart. Low-prognosis women were classified into four groups as per the POSEIDON classification, with the control group consisting of normal and good responders

The overall study flow diagram is shown in Fig. 1. In total, 17,698 patients were separated into five groups: POSEIDON group 1 (younger unexpected poor responders, *n* = 4470), POSEIDON group 2 (older unexpected poor responders, *n* = 2270), POSEIDON group 3 (younger expected poor responders, *n* = 1110), POSEIDON group 4 (older expected poor responders, *n* = 1095), and group 5 (control group, *n* = 8753). Patients in the control group exhibited a sufficient ovarian reserve (AFC ≥ 5) and a sufficient number of oocytes retrieved (> 9) during the first ovarian retrieval cycle. Patient demographics and baseline characteristics are shown in Table 1.

**Table 1:** Demographics and baseline characteristics of the participants in the first oocyte retrieval cycle

Group	POSEIDON group				Control group	***P*** value
	Group1	Group2	Group3	Group4	Group5	
	Younger unexpected poor responders	Older unexpected poor responders	Younger expected poor responders	Older expected poor responders		
**Patients (n)**	4470	2270	1110	1095	8753	
**Age of female**	30.21 ± 2.76^a^	38.01 ± 2.83^b^	30.32 ± 2.71^a,d^	39.37 ± 3.38^c^	30.44 ± 3.85^d^	<0.05
**Percentage of age**	a	b	c	d	e	<0.05
≤ 30	2234 (50.0%)	0 (0.0%)	541 (48.7%)	0 (0.0%)	4760 (54.4%)	
> 30, ≤35	2236 (50.0%)	494 (21.8%)	569 (51.3%)	141 (12.9%)	3103 (35.5%)	
> 35, ≤40	0 (0.0%)	1360 (59.9%)	0 (0.0%)	564 (51.5%)	796 (9.1%)	
> 40	0 (0.0%)	416 (18.3%)	0 (0.0%)	390 (35.6%)	94 (1.1%)	
**AFC**	11.17 ± 5.77^a^	8.99 ± 4.09^b^	2.19 ± 1.65^c^	2.51 ± 1.32^c^	15.24 ± 6.21^d^	<0.05
**Percentage of AFC**	a	b	c	c	d	<0.05
< 4	0 (0.0%)	0 (0.0%)	750 (67.6%)	776 (70.9%)	0 (0.0%)	
≥ 4, < 10	2185 (48.9%)	1502 (66.2%)	360 (32.4%)	319 (29.1%)	1444 (16.5%)	
≥ 10	2285 (51.1%)	768 (33.8%)	0 (0.0%)	0 (0.0%)	7309 (83.5%)	
**Oocytes retrieved in the first cycle (n)**	5.97 ± 2.31^a^	5.27 ± 2.34^b^	7.07 ± 6.67^c^	3.29 ± 3.05^d^	16.84 ± 6.57^e^	<0.05
**Type of infertility**	a	b	c	d	e	<0.05
Primary	2673 (59.8%)	848 (37.4%)	713 (64.2%)	383 (35.0%)	5059 (57.8%)	
Secondary	1797 (40.2%)	1422 (62.6%)	397 (35.8%)	712 (65.0%)	3694 (42.2%)	
**Main etiology**	a	b	c	d	e	<0.05
Female	460 (10.3%)	240 (10.6%)	94 (8.5%)	69 (6.3%)	1076 (12.3%)	
Male	2945 (65.9%)	1401 (61.7%)	770 (69.4%)	741 (67.7%)	5567 (63.6%)	
Combined	539 (12.0%)	236 (10.4%)	126 (11.3%)	132 (12.0%)	1059 (12.1%)	
Others	526 (11.8%)	393 (17.3%)	120 (10.8%)	153 (14.0%)	1051 (12.0%)	
**Duration of infertility (years)**	2.93 ± 2.42^a^	4.08 ± 3.96^b^	3.14 ± 2.46^c^	4.32 ± 4.56^d^	3.01 ± 2.70^a,c^	<0.05
**Female BMI (kg/m**^**2**^**)**	22.00 ± 4.74^a^	22.26 ± 3.07^b^	21.78 ± 5.87^a,c^	22.16 ± 2.88^a,b^	21.64 ± 3.31^c^	<0.05
**Year of treatment**	a	b	c	d	e	<0.05
2013–2014	1160 (26.0%)	526 (23.2%)	309 (27.8%)	304 (27.8%)	2248 (25.7%)	
2015	859 (19.2%)	375 (16.5%)	258 (23.2%)	180 (16.4%)	1821 (20.8%)	
2016	1349 (30.2%)	730 (32.2%)	362 (32.6%)	348 (31.8%)	2188 (25.0%)	
2017	683 (15.3%)	353 (15.6%)	111 (10.0%)	172 (15.7%)	1353 (15.5%)	
2018	419 (9.4%)	286 (12.6%)	70 (6.3%)	91 (8.3%)	1143 (13.1%)	
**Gravidity**	0.71 ± 1.27^a^	1.42 ± 1.53^b^	0.62 ± 1.04^c^	1.48 ± 1.52^b^	0.74 ± 1.09^a^	<0.05
**Parity**	0.06 ± 0.26^a^	0.27 ± 0.48^b^	0.05 ± 0.23^a^	0.30 ± 0.51^b^	0.08 ± 0.30^c^	<0.05

Values are presented as mean ± SD or number (percentage). The differences were considered statistically significant when the *p*-value was less than 0.05. Different alphabets represent significant differences between groups. *AFC* Antral follicle count, *BMI* Body mass index

^a,b,c,d,e^ *P*<0.05

When patients were grouped according to POSEIDON criteria (Table 1), the average ages of the older patients in group 2 (38.01 ± 2.83) and group 4 (39.37 ± 3.38) were notably higher than those of younger patients in group 1 (30.21 ± 2.76), group 3 (30.32 ± 2.71), and the control group (30.44 ± 3.85). Consistent with grouping criteria, the average AFC of expected poor responders in group 3 (2.19 ± 1.65) and group 4 (2.51 ± 1.32) was significantly lower than that of group 1 (11.17 ± 5.77), group 2 (8.99 ± 4.09), and control group patients (15.24 ± 6.21). Average numbers of oocytes retrieved during the first cycle were highest in the control group (16.84 ± 6.57), followed by group 3 (7.07 ± 6.67), group 1 (5.97 ± 2.31), group 2 (5.27 ± 2.34), and group 4 (3.29 ± 3.05). Owing to heterogeneity among low-prognosis women, types of infertility, primary etiology, and BMI all differed significantly among these five groups (*P < 0.05*). Furthermore, with increasing age, the duration of infertility rose group 1 (2.93 ± 2.42) to group 3 (3.14 ± 2.46), group 2 (4.08 ± 3.96), and group 4 (4.32 ± 4.56). Gravidity, parity, and year of treatment also differed significantly among these groups (*P* < 0.05).

### Ovarian stimulation characteristics and pregnancy outcomes

As expected, patients in group 4 (older expected poor responders) underwent the most oocyte retrieval cycles (1.97 ± 1.33), followed by patients in group 3 (younger expected poor responders, 1.55 ± 0.94), group 2 (older unexpected poor responders, 1.54 ± 0.89), and group 1 (younger unexpected poor responders, 1.33 ± 0.64), as shown in Table 2. Patients in the control group underwent the fewest oocyte retrieval cycles (1.11 ± 0.38) (*P* < 0.05). However, the number of retrieved oocytes was smallest in group 4 (3.29 ± 3.05), and increased in group 2 (5.27 ± 2.34), group 1 (5.97 ± 2.31), and group 3 (7.07 ± 6.67), with the greatest number of oocytes having been retrieved for patients in the control group (16.84 ± 6.57) (*P* < 0.05). As such, the oocyte output rate (number of oocytes retrieved / AFC × 100%) [19] was highest in group 3 (younger expected poor responders, 199.21%), followed by group 5 (normal or high responders, 109.28%), group 4 (older expected poor responders, 99.31%), group 2 (older unexpected poor responders, 62.96%), and group 1 (younger unexpected poor responders, 58.02%). Patients in the control group also exhibited the highest number of 2PN (10.39 ± 5.09) and good quality embryos (5.32 ± 3.78) per oocyte retrieval cycle, followed by patients in group 3 (4.47 ± 4.26; 2.38 ± 2.59), group 1 (3.86 ± 2.08; 2.15 ± 1.71), group 2 (3.44 ± 1.95; 1.98 ± 1.55), and group 4 (2.16 ± 2.13; 1.27 ± 1.39) (*P* < 0.05). Compared with other groups, POSEIDON group 4 had more one embryo transferred (23.9%). The control group had the highest percentage of two embryos transferred (62.9%). As for the type of embryos transferred, the proportions for D3 embryos were 84.3, 87.3, 85.4, 88.0 and 84.0%, respectively, and the proportions among Day 5/6 embryos transferred were 15.7, 12.7, 14.6, 12.0 and 16.0% from group 1 to 5.

**Table 2:** Ovarian Stimulation Characteristics and Cumulative live birth rates within POSEIDON and control groups

	POSEIDON group				Control group	***P***-value
	Group1	Group2	Group3	Group4	Group5	
	Younger unexpected poor responders	Older unexpected poor responders	Younger expected poor responders	Older expected poor responders		
**Patients (n)**	4470	2270	1110	1095	8753	
**Oocyte retrieval cycles (n)**	1.33 ± 0.64^a^	1.54 ± 0.89^b^	1.55 ± 0.94^b^	1.97 ± 1.33^c^	1.11 ± 0.38^d^	<0.05
**Retrieved oocytes (n)**	5.97 ± 2.31^a^	5.27 ± 2.34^b^	7.07 ± 6.67^c^	3.29 ± 3.05^d^	16.84 ± 6.57^e^	<0.05
**Retrieved Oocytes / AFC (%)**	58.02%	62.96%	199.21%	99.31%	109.28%	<0.05
**Cycles of 0 oocyte Retrieved (%)**	25 (0.4%)^a^	12 (0.3%)^a^	32 (1.9%)^b^	51 (2.4%)^b^	0 (0.0%)^c^	<0.05
**Insemination method**	a	b	c	d	e	<0.05
IVF	2878 (64.6%)	1486 (65.8%)	781 (70.9%)	752 (69.8%)	5069 (58.0%)	
ICSI	988 (22.2%)	572 (25.3%)	235 (21.3%)	290 (26.9%)	2047 (23.4%)	
IVF/ICSI	587 (13.2%)	201 (8.9%)	86 (7.8%)	36 (3.3%)	1630 (18.6%)	
**2PN embryos per oocyte retrieval cycle (n)**	3.86 ± 2.08^a^	3.44 ± 1.95^b^	4.47 ± 4.26^c^	2.16 ± 2.13^d^	10.39 ± 5.09^e^	<0.05
**Good quality embryos per oocyte retrieval cycle (n)**	2.15 ± 1.71^a^	1.98 ± 1.55^b^	2.38 ± 2.59^c^	1.27 ± 1.39^d^	5.32 ± 3.78^e^	<0.05
**FET cycles (n)**	1.46 ± 0.81^a^	1.58 ± 0.92^b^	1.47 ± 0.78^a,b^	1.54 ± 0.94^a,b^	1.57 ± 0.88^b^	<0.05
**Transplanted embryos (n)**	2.31 ± 1.61^a^	2.50 ± 1.88^b^	2.27 ± 1.70^a^	2.01 ± 1.74^c^	3.08 ± 1.88^d^	<0.05
**CLBR over 2 years**						
Optimistic(95%CI)	0.76 (0.74–0.77)^a^	0.55 (0.53–0.58)^b^	0.67 (0.63–0.70)^c^	0.36 (0.32–0.39)^d^	0.76 (0.75–0.77)^e^	<0.001
Conservative(95%CI)	0.64 (0.62–0.65)^a^	0.42 (0.40–0.44)^b^	0.55 (0.52–0.58)^c^	0.26 (0.23–0.29)^d^	0.70 (0.70–0.71)^e^	<0.001
**CLBR over 5 years**						
Optimistic(95%CI)	0.81 (0.79–0.82)^a^	0.60 (0.57–0.63)^b^	0.75 (0.71–0.78)^c^	0.41 (0.37–0.46)^d^	0.79 (0.78–0.80)^a^	<0.001
Conservative(95%CI)	0.66 (0.65–0.67)^a^	0.44 (0.42–0.46)^b^	0.58 (0.55–0.61)^c^	0.27 (0.25–0.30)^d^	0.72 (0.72–0.73)^e^	<0.001

^a,b,c,d,e^ *P*<0.05

Table 2 displays the CLBRs within 2 and 5 years in each group with corresponding 95% CIs. When using the optimistic method, the CLBRs over 2 years were 75.6, 55.5, 67.2, 35.7, and 76.2% in groups 1–5, respectively. Using the conservative method, the CLBRs over 2 years in groups 1–5 were 63.7, 42.1, 55.3, 25.9, and 70.5%, respectively. When the CLBR timeframe was extended to 5 years, similar decreasing trends were observed from group 5 (optimistic: 79.3%; conservative: 72.5%) to group 1 (80.8%; 66.0%), group 3 (75.0%; 58.4%), group 2 (60.3%; 43.6%), and group4 (41.4%; 27.2%). However, there may be differences in the extent of these CLBRs among these groups over the entirety of this 5-year period. As such, we utilized time-dependent CLBR curves to trace the extent of CLBR improvements in each POSEIDON subgroup.

### Cumulative live birth rates within 5 years

**Fig. 2:**
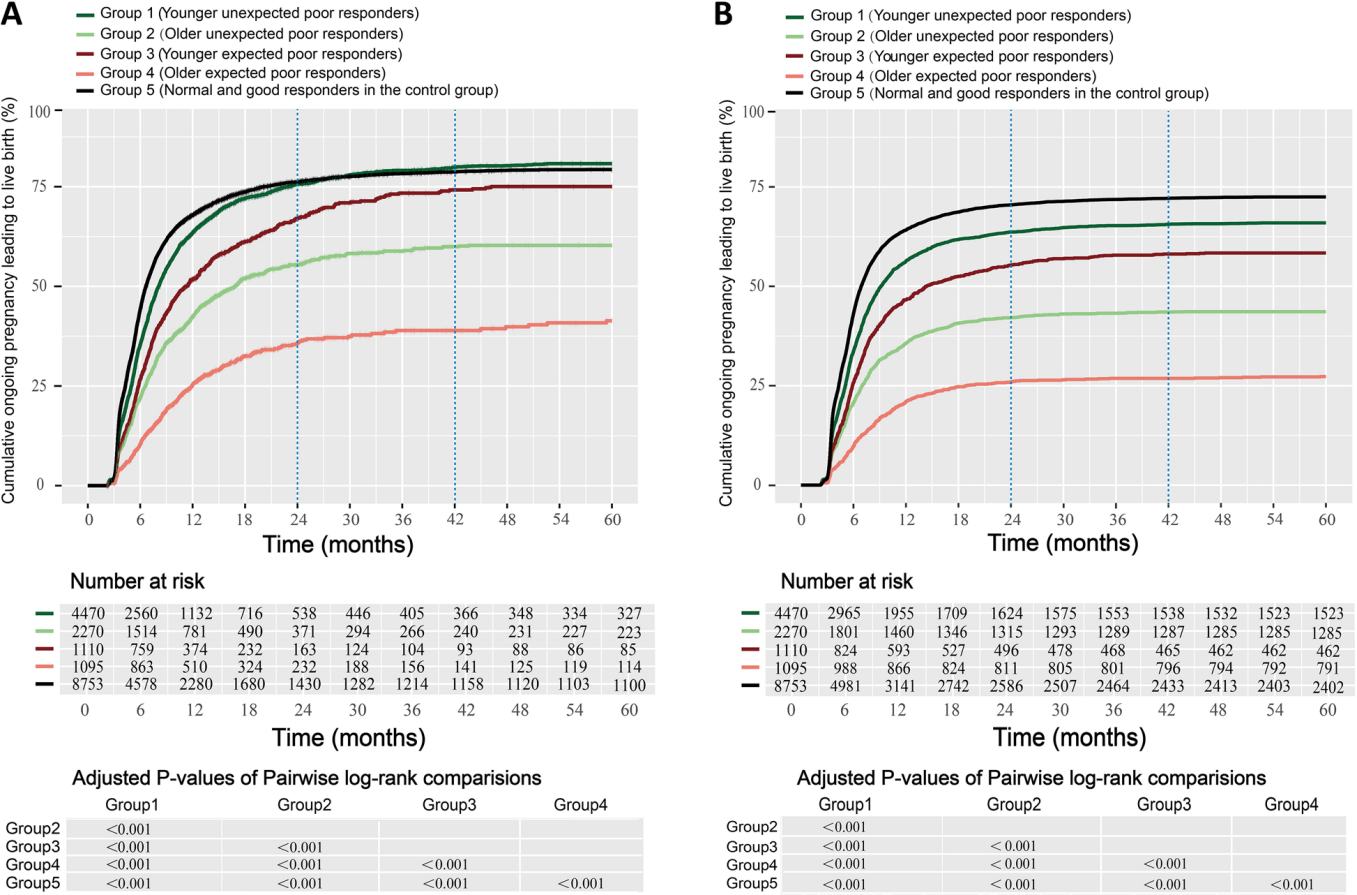
Cumulative live birth curves for low-prognosis women over 5 years. Two approaches were applied to depict cumulative live birth curves over 5 years: (**A**) an optimistic method (a life table analysis) and (**B**) a conservative method (a competing risk analysis). The number of risks and the pairwise comparisons between different POSEIDON groups are listed below the CLBR curves

The cumulative incidence curves and pairwise log-rank comparisons for CLBRs between the optimistic and conservative methods in these five groups are shown in Fig. 2. All curves differed significantly among these five groups (*P* < 0.001). Under both the optimistic and conservative analytical methods, patients in all groups exhibited rapid increases in CLBRs within the first 1–2 years, with the CLBR of group 5 (control group) being the highest (optimistic: 76.2%; conservative: 70.5%), followed by that of group 1 (younger unexpected poor responders, optimistic: 75.6%; conservative: 63.7%), group 3 (younger expected poor responders, optimistic: 67.2%; conservative: 55.3%), group 2 (*older unexpected* poor responders, optimistic: 55.5%; conservative: 42.1%), and group 4 (*older expected poor responders*, optimistic: 35.7%; conservative: 25.9%).

Over the following 2- to 3.5-year periods, CLBRs in these five groups remained in the same rank-order and rose modestly when analyzed via the optimistic method (control group: 2.6%; group 1: 4.5%; group 3: 7.3%; group 2: 4.6%; group 4: 3.2%), whereas they rose more slowly when assessed via the conservative method (control group: 1.6%; group 1: 1.9%; group 3: 2.8%; group 2: 1.4%, group 4: 0.9%). Within 3.5–5 years, CLBRs in all five groups largely plateaued (control group: 0.5%; group 1: 0.7%; group 3: 0.5%; group 2: 0.2%, group 4: 2.5% with the optimistic method; control group: 0.4%; group 1: 0.4%; group 3: 0.3%; group 2: 0.1%, group 4: 0.4% with the conservative method).

Notably, among these five groups the CLBRs of patients in group 3 (younger expected poor responders increased most significantly within 2–3.5 years under both the optimistic (7.3%) and conservative (2.8%) analytical methods. Interestingly, the CLBRs of patients in group 4 (*older expected poor responders) also rose notably within the atter 3.5–5-year period (optimistic: 2.5%; conservative: 0.4%), whereas the other four groups had largely plateaued by this time point. As the interval between oocyte retrievals or FET cycles differed among patients, this may not accurately reflect upward trends in CLBRs over time among groups. As such, we conducted further analyses of CLBR improvements as a function of the number of FET cycles completed in each of these* POSEIDON subgroups.

### Cumulative live birth rates within 9 FET cycles

**Fig. 3:**
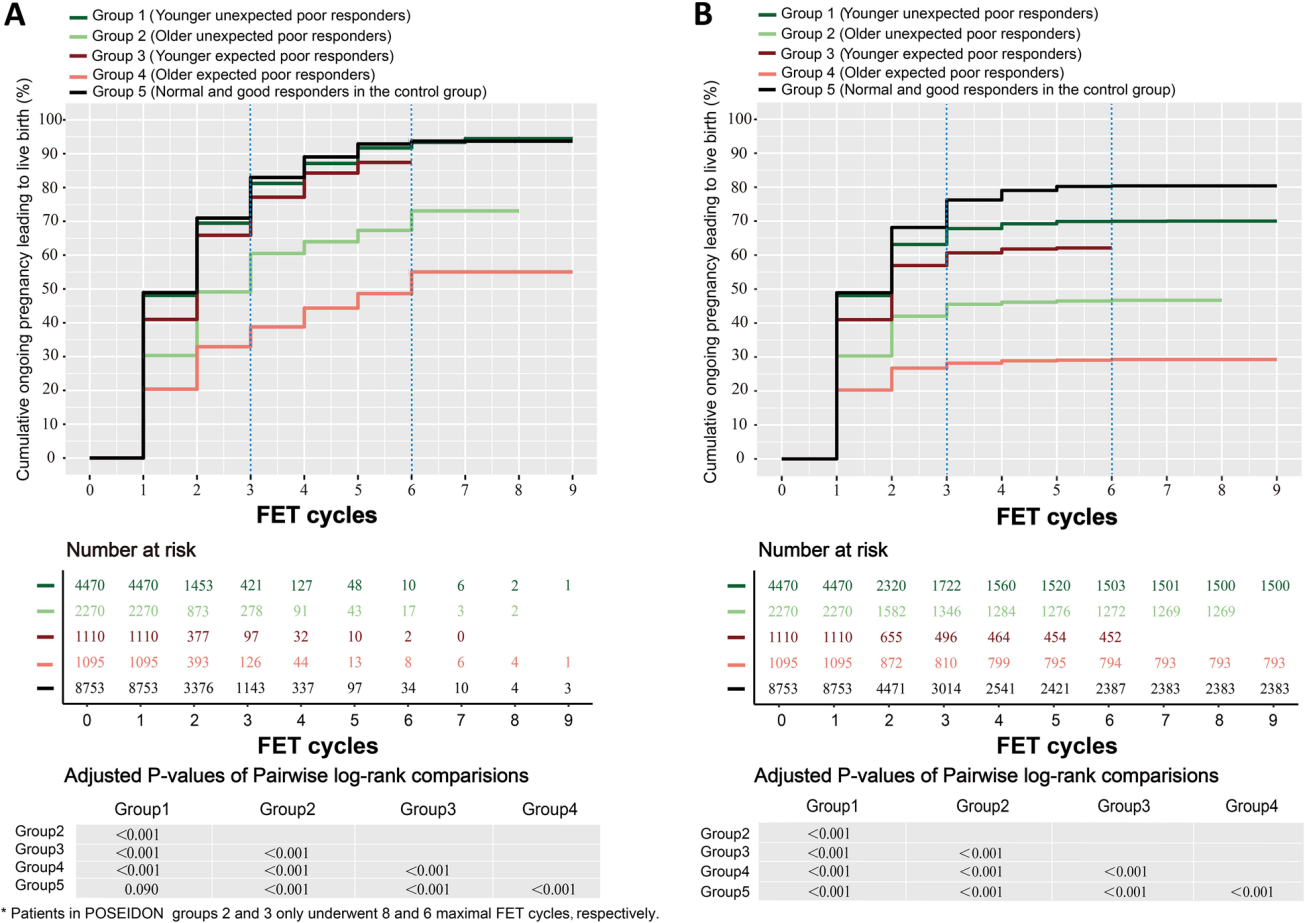
Cumulative live birth curves for low-prognosis women over 9 FET cycles. Two approaches were applied to depict cumulative live birth curves over 9 FET cycles: (**A**) an optimistic method (a life table analysis) and (**B**) a conservative method (a competing risk analysis). The number of risks and the pairwise comparison between different POSEIDON groups are listed below the CLBR curves

Cumulative ongoing birth rates as a function of the number of FET cycles completed, as analyzed via both the conservative and optimistic approaches, are shown in Fig. 3. CLBR curves differed significantly among groups with the exception of those for groups 1 and 5 (*P* < 0.001). Under both the optimistic and conservative models, patients in these five groups exhibited rapid initial increases in CLBRs within 3 FET cycles, with these CLBRs decreasing from group 5 (control group, optimistic: 83.0%; conservative: 76.2%) to group 1 (younger unexpected poor responders, optimistic: 81.2%; conservative: 67.8%), group 3 (younger expected poor responders, optimistic: 77.1%; conservative: 60.7%), group 2 (*older unexpected* poor responders, optimistic: 60.5%; conservative: 45.5%), to group 4 (*older expected poor responders*, optimistic: 38.8%; conservative: 28.2%).

From 4 to 6 FET cycles, CLBRs in these five groups remained in the same rank-order as above and exhibited modest increases under the optimistic method (control group: 10.7%; group 1: 12.1%; group 3: 10.3% (only 5 FET cycles); group 2: 12.6%; group 4: 16.3%). While in the conservative method, the increase of CLBRs within five groups were extremely slowly (control group: 4.2%; group 1: 2.2%; group 3: 1.4%; group 2: 1.2%; group 4: 1.1%). Then CLBRs of five groups all maintained in their plateaus after 6 FET cycles, no matter in the optimistic or conservative methods.

## Discussion

For the low-prognosis women, LBR of per embryo transfer cycle or CLBR of all embryos retrieved in the first IVF/ICSI cycle could not reflect their chance of having a live birth in a comprehensive manner. In contrast, the CLBR analyses conducted in this study offer insight into outcomes throughout the entire course of ART treatment, providing more robust guidance for different POSEIDON subgroups of low-prognosis women. Overall, we found that CLBRs for low-prognosis women declined from group 1 (younger unexpected poor responders) to group 3 (younger expected poor responders), group 2 (older unexpected poor responders), and group 4 (older unexpected poor responders) as a function of treatment time or number of FET cycles under both conservative and optimistic analytical methods. While the specific CLBRs in these four POSEIDON subgroups exhibited different trajectories, all low-prognosis women exhibited increases in CLBRs over the first 2 years or 3 FET cycles, and these rates continued to rise moderately from 2 to 3.5 years or 4–6 FET cycles. After 3.5 years or 6 FET cycles, the CLBRs of all analyzed low-prognosis women largely plateaued. Together, our findings offer insight into the CLBRs of patients in each POSEIDON subgroup over 5 years and 9 FET cycles, providing a valuable reference for clinicians undergoing ART treatment.

### Strengths and limitations

To the best of our knowledge, this study traced the long-term CLBRs of different groups of low-prognosis patients over the longest timespan and the highest number of FET cycles. The CLBR used in this study encompassed all oocyte retrievals and subsequent FET cycles, rather than the pregnancy outcome of the first fresh or frozen embryo transfer cycle [47], with this being distinct from previously reported CLBRs that were only associated with a single oocyte retrieval cycle [19]. As such, the CLBR used herein is more comprehensive and objective as a means of assessing pregnancy outcomes among low-prognosis women. Moreover, our application of both optimistic and conservative approaches ensures that our findings will remain robust while appropriately addressing the issue of treatment discontinuation [45].

We additionally explored improvements in CLBR as a function of treatment duration and FET cycle count in different groups of low-prognosis women with heterogeneous characteristics that had been grouped in accordance with POSEIDON criteria. This approach is a powerful strategy capable of offering insight into whether or not indefinite ART treatment will continue to improve CLBRs for these low-prognosis women. Overall, our results demonstrate that all low-prognosis women in all four POSEIDON groups should undergo ART treatment for a minimum of 2 years or 3 FET cycles, and were likely to achieve better outcomes when extending treatment durations to 3.5 years or 6 FET cycles, particularly for women in group 3 (younger expected poor responders) and group 4 (older expected poor responders). However, no groups appeared to benefit substantially from undergoing ART treatment for more than 3.5 years or 6 FET cycles, suggesting that these guidelines can be used to guide counseling and consultation for different groups of low-prognosis women in order to maximize their odds of a live birth while reducing unnecessary and unproductive expenditures.

Despite the above mentioned advantages of our study, it is nonetheless subject to certain limitations. For one, this was a single-center retrospective study and the studied patients only adopted PPOS approcach as the ovarian stimulation protocol. While a large number of FET cycles for low-prognosis women were included in these analyses, the retrospective nature of this research ensures that further validation will be required to confirm these conclusions. Additionally, we did not assess anti-Mullerian hormone (AMH), which is also a predictor of ovarian response, when categorizing women into the expected and unexpected poor responder categories as these data were not present in our database. The discrimination of low-prognosis women by POSEIDON criteria partly depends on the number of oocytes retrieved, which requires at least one previous oocyte retrieval cycle. Unexpected poor responders cannot be identified immediately when it comes to clinical judgment. Moreover, patients in group 3 (younger expected poor responders) only routinely underwent 5 FET cycles in this study, preventing us from fully assessing their improvements in CLBRs in the 4–6 FET cycle range, particularly given that patients in group 4 (older expected poor responders) exhibited striking improvements in CLBRs within 4–6 FET cycles.

### Differences between our results and other research

Prior studies of low-prognosis women have often utilized primary outcome indicators such as live birth rates per embryo transfer cycle or cumulative pregnancy rates from a single oocyte retrieval cycle [2, 19, 48, 49]. However, for low-prognosis women that exhibit low pregnancy rates [2] and typically require more than one cycle to achieve a live birth [50], the odds of having a baby at any point during the ART treatment process are the most important endpoint of interest. As such, our study adopted cumulative pregnancy rates over multiple ART cycles including all oocyte retrievals and subsequent FET cycles [50, 51], thus emphasizing the overall pregnancy rates given that these are the most important endpoint for this low-prognosis patient population.

We additionally explored whether low-prognosis patients should continue pursuing ART indefinitely as a means of improving CLBRs by tracing improvements in CLBRs over time or with increases in FET cycle count. Treatment times as long as 5 years were included, as they included all episodes of live birth (including second or greater births) associated with a single round of egg collection [3]. It is also important that low-prognosis patients consider how best to balance the completion of multiple rounds of oocyte retrieval for banking purposes with the timely transplantation of fertilized embryos [3, 4]. As such, we additionally explored changes in CLBRs with increasing numbers of FET cycles (up to 9 FET cycles). In so doing, we sought to address the question of when clinicians should advise low-prognosis patients to cease undergoing further ART attempts, as prior studies have been unable to establish such a threshold owing to their short duration [45, 50].

Low-prognosis patients exhibit substantial heterogeneity with respect to their ultimate outcomes, largely owing to differences in age, ovarian reserve test cut-off values, and other risk factors [25, 52–54]. For this study, we utilized the POSEIDON criteria to stratify patients into four groups as a means of reducing such heterogeneity and better defining optimal ART treatment strategies for each of these patient subgroups. Based upon the observed increases in CLBRs over time and with increasing numbers of FET cycles, we recommend that low-prognosis patients in all POSEIDON subgroups extend their treatment for no more than 3.5 years or 6 FET cycles, with a minimum treatment duration of greater than 2 years or 3 FET cycles. In addition, patients in group 3 (younger expected poor responders) and group 4 (older expected poor responders) exhibited particularly pronounced benefits when extending the duration of treatment to 3.5 years or 6 FET cycles.

### Mechanisms underlying different cumulative live birth rates in low-prognosis women

Age is the primary determining factor for CLBR among low-prognosis women. In our data, patients in group 1 (30.21 ± 2.76) and group 3 (30.32 ± 2.71) exhibited significantly higher CLBRs relative to those of the older patients of group 2 (38.01 ± 2.83) and group 4 (39.37 ± 3.38), irrespective of time or FET cycle count. The age threshold of 35 years is generally considered to represent a watershed with respect to changes in embryo quality and quantity. As women age, rates of embryo euploidy fall by 2.4% per year, while rates of blastocyst euploidy fall from 60% prior to age 35 to 30% after age 40, with corresponding reductions in the odds of implantation [55, 56]. As such, after 5 FET cycles or 3.5 years, the optimal estimated CLBRs for young POSEIDON group 1 (91.7, 80.1%) and group 3 (87.4, 74.5%) patients were similar to those of normal women (92.9% or 78.8%), whereas these rates were substantially lower for old er POSEIDON group 2 (67.3, 60.1%) and group 4 (48.6, 38.9%) patients..

Within patients of a similar age, ovarian reserve is the second most important determinant of CLBR among low-prognosis women. Variations in CLBR between POSEIDON patient subgroups are secondarily attributable to the quantitative parameters [45], in line with reports demonstrating that lower AMH levels and decreased ovarian responses are related to lower odds of a live birth among women of similar age [57–59]. As such, while patients in both groups 1 and 3 were under 35 years of age, the CLBR of patients in group 1 (unexpected poor responders) was significantly higher than that of patients in group 3 (expected poor responders). Similar phenomena have been observed in the older POSEIDON groups, with patients in group 2 (unexpected poor responders) exhibiting a higher CLBR than that of patients in group 4 (expected poor responders) irrespective of the duration of time or number of FET cycles.

Recent work also suggests that CLBRs rise significantly with the number of retrieved oocytes [60]. This likely explained the observed increases in CLBRs in all four POSEIDON groups over 5 years or increasing numbers of FET cycles in this study, irrespective of patient age or ovarian reserve. However, there was substantial heterogeneity among these four POSEIDON groups, exhibiting differing levels of increases in CLBRs over time or with increasing FET cycles. Within 2–3.5 years or 4–6 FET cycles, the CLBRs in group 3 (younger expected poor responders) and group 4 (older expected poor responders) exhibited clearer increases in CLBRs relative to the other POSEIDON groups, likely owing to oocyte accumulation and effects being more substantially increased in these expected poor responders.

## Conclusions

In conclusion, we herein conducted an analysis of CLBRs for women in different POSEIDON groups and controls over the course of up to 5 years or 9 FET cycles in order to provide a better clinical reference for low-prognosis women undergoing ART treatment. Patients in group 1 (younger unexpected poor responders) exhibited the highest CLBR within the 5-year or 9 FET cycle period, followed by group 3 (younger expected poor responders), group 2 (older unexpected poor responders), and group 4 (older unexpected poor responders) patients. While there were differences in CLBRs among these groups, the observed upward trends suggest that all low-prognosis women should undergo ART treatment for a minimum of 2 years or 3 FET cycles, with better outcomes over 3.5 years or 6 FET cycles, particularly for group 3 and group 4 patients. However, beyond those milestones, patients should consider ceasing to undergo further ART treatment. Together, these data provide new insight into how long low-prognosis women should seek to undergo ART treatment in an effort to improve their odds of a live birth.

## Data Availability

The datasets used and/or analysed during the current study available from the corresponding author on reasonable request.

## Abbreviations

POR: Poor ovarian response
DOR: Diminished ovarian reserve
ART: Assited reproductive technology
CLBR: Cumulative live birth rate
ET: Fresh embryo transfer
FET: Frozen embryo transfer
ESHRE: European Society of Human Reproduction and Embryology
POSEIDON: Patient-Oriented Strategies Encompassing Individualized Oocyte Number
IVF: In vitro fertilization
ICSI: Intracytoplasmatic sperm injection
AFC: Antral follicle count
MPA: Medroxyprogesterone acetate
DYG: Duphaston
SD: Standard deviation
OPU: Ovum pick-up
PPOS: Progestin-primed ovarian stimulation
BMI: Body mass index
CI: Confidential interval
LBR: Live birth rate
AMH: Anti miillerian hormone
